# Pakistan's COVID-19 Prevention and Control Response Using the World Health Organization's Guidelines for Epidemic Response Interventions

**DOI:** 10.7759/cureus.34480

**Published:** 2023-01-31

**Authors:** Faran Emmanuel, Anusheh Hassan, Ahsan Ahmad, Tahira E Reza

**Affiliations:** 1 Epidemiology and Public Health, University of Manitoba, Winnipeg, CAN; 2 Public Health, Institute of Global Public Health, University of Manitoba, Winnipeg, CAN; 3 Epidemiology and Public Health, Health Planning, System Strengthening and Information Analysis Unit, Islamabad, PAK; 4 Epidemiology and Biostatistics, Centre for Global Public Health (CGPH-Pakistan), Islamabad, PAK

**Keywords:** guidelines, who, response, covid-19, pakistan

## Abstract

Massive coronavirus disease 2019 (COVID-19) devastation was anticipated in Pakistan due to poor track record of responding to epidemics. However, by adopting effective and timely response measures under strong government leadership, Pakistan averted a significant number of infections.

We present the government of Pakistan’s efforts to curb the spread of COVID-19, using the World Health Organization’s guidelines for epidemic response intervention. The sequence of interventions is presented under the epidemic response stages, namely anticipation, early detection, containment-control, and mitigation. Key factors of Pakistan’s response included decisive political leadership and implementation of a coordinated and evidence-informed strategy. Moreover, early control measures, mobilization of front-line health workers for contact tracing, public awareness campaigns, 'smart lockdowns', and massive vaccination drives are key strategies that helped flatten the curve. These interventions and lessons learnt can help countries and regions struggling with COVID-19 to develop successful strategies to flatten the curve and enhance disease response preparedness.

## Introduction and background

The coronavirus disease 2019 (COVID-19) pandemic has witnessed an unprecedented global health crisis, the magnitude of which has impacted countries with far-reaching economic and social consequences [1]. It has pushed healthcare systems beyond their optimal functional limits, highlighting huge inequities in health and the critical importance of having resilient health systems [2,3]. The impact of COVID-19 on people has been largely influenced by the manner in which countries have responded to the pandemic [4]. While countries such as South Korea, New Zealand, Thailand and Pakistan were successful in limiting the early spread of the disease, other countries like the United States, Brazil, and India had less successful outcomes [5,6].

**Figure 1:**
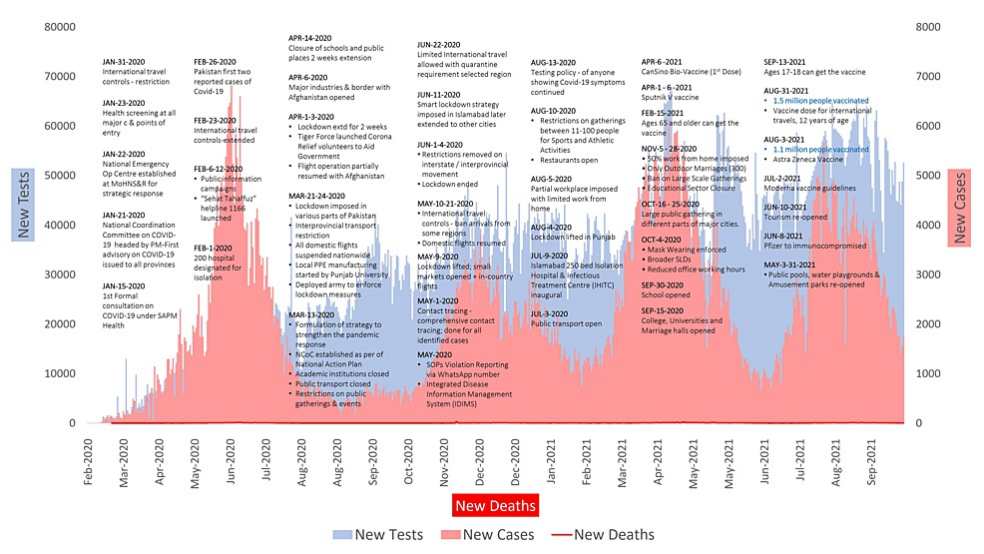
COVID-19 epidemic curve for Pakistan (March 2020 to September 2021) accounting for four waves. This is an original figure for this research

Due to high population density, significant healthcare challenges, and a poor track record of responding to infectious diseases such as polio, hepatitis and tuberculosis, massive COVID-19-related devastation was anticipated in Pakistan [7,8]. However, by quickly implementing effective response measures under strong government leadership, Pakistan was able to avert a substantial number of COVID-19 infections. Pakistan recorded its first two COVID-19 cases on February 26, 2020. The epidemic started to proliferate and the first case of community transmission was documented on March 29, 2020. The daily case number began to surge, reaching the highest number of 6,825 cases on June 13, 2020. The number of deaths also peaked during May and June 2020 with the highest number of daily deaths recorded during the second and third weeks of June. The number of daily cases gradually started to decline in July and this trend continued through August and September 2020. The COVID-19 test positivity rates correspondingly declined from a peak positivity rate of 22.2% in June to 1.7% in September 2020 (Figure 1) [9]. Subsequent waves were observed from mid-October 2020 to mid-February 2021 (second wave), and mid-February 2021 to mid-June 2021 (third wave). The number of confirmed cases ranged between 242,200 to 379,988 across the respective waves with a case fatality ratio ranging between 2.06 to 2.35% (Table 1).

**Table 1:** COVID-19 waves in Pakistan (March 2020 to September 2021).

	Wave Time Frame	Confirmed Cases	Deaths	Case Fatality Rate	Number of tests
First wave	Mar 20 to Mid Oct 20	321,877	6,621	2.06%	3.98 million
Second wave	Mid Oct 20 to Mid-Feb 21	242,200	5,712	2.19%	4.49 million
Third wave	Mid-Feb 21 to Mid-June 2021	379,988	9,448	2.35%	5.42 million
Fourth wave	Mid- Jun 21 – Sep 21	309,803	6,175	2.23%	5.8 million

In this paper, we describe Pakistan’s prevention and control plan to respond to the COVID-19 pandemic, as well as analyze the response using the World Health Organization’s (WHO) guidelines for epidemic response interventions, specifically anticipation, early detection, containment, and control and mitigation [10,11].

## Review

National coordination and leadership

**Figure 2:**
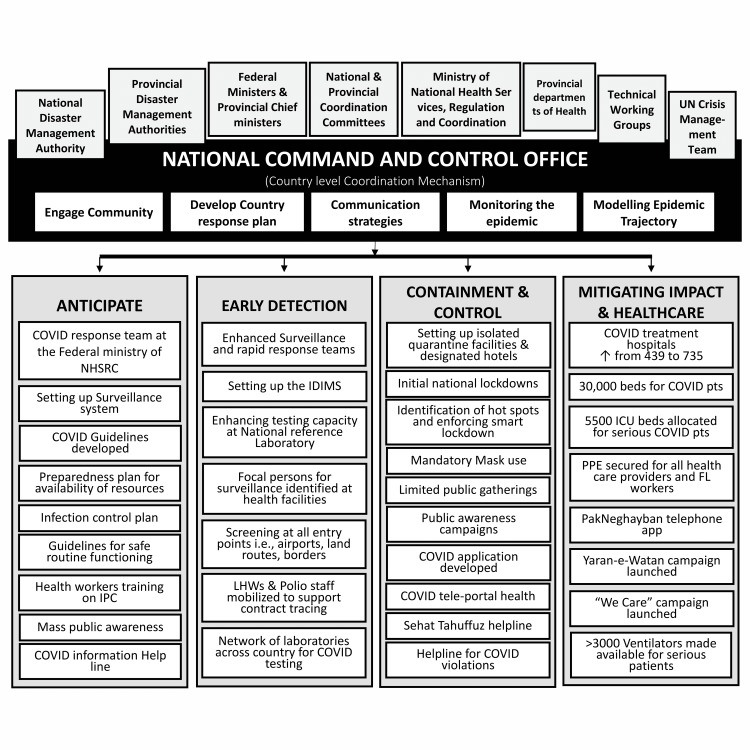
National coordination and a summary of response with various epidemic stages. LHWs: lady health workers, IDIMS: Integrated Disease Information Management System, NHSRC: National Health Services Regulation and Coordination, IPC: infection prevention control, PPE: personal prevention equipment This is an original figure

Addressing a crisis as huge as COVID-19 necessitated the highest level of political commitment to implement a well-organized and coordinated response based on a clear scientific strategy. The federal government was quick to take on the leadership role and mobilize relevant stakeholders to synergize and articulate a unified national response against COVID-19. The National Security Committee, chaired by the Prime Minister of Pakistan, established a National Coordination Committee (NCC). The NCC was chaired by the Prime Minister and comprised all relevant federal ministers, chief ministers of all provinces and provincial health departments. It designated the National Disaster Management Authority (NDMA) as the leading operational agency and created provincial task forces chaired by the respective chief ministers. Moreover, a National Command Operation Centre (NCOC) was established in March 2020 to ensure effective coordination between the federal and provincial governments. The NCOC collated, analyzed and processed all COVID-19-related data, and served as the nerve center to synergize and articulate a unified national effort against COVID-19 and implement decisions of the NCC (Figure 2). A Crisis Management Team (CMT) comprising WHO and all UN bodies was also mobilized to coordinate the UN response in Pakistan [12,13]. Pakistan’s overall response to COVID-19 was based on the guidance of the WHO Strategic and Technical Advisory Group for Infectious Hazards, which recommends that all countries should consider a combination of response measures [10]. The response and the sequence of interventions are described below using WHO’s recommended stages of epidemic response [14].

The 'anticipation' stage

The severity of the outbreaks in neighboring countries such as China and Iran prompted the government to implement immediate precautionary measures much before the first cases were reported in Pakistan. A COVID-19 response team was formulated at the Ministry of National Health Services, Regulations and Coordination (M/o NHSR&C) in early January 2021, that quickly reviewed national capacity in the area of detection and response, and devised a strategic preparedness plan based on the WHO’s scientific guidance [9,15]. The Ministry initialized a surveillance and real-time data collection system, ensured provision of personal prevention equipment (PPE) and other infection prevention control (IPC) supplies, quickly set up isolation facilities, and allocated hospital beds for rapid availability of treatment for patients who may get affected. A number of guidelines for safe functioning of routine activities were formulated and healthcare workers were trained on infection prevention and control protocols. The National Reference Public Health Laboratory quickly acquired the requisite capability for COVID-19 diagnostics on February 1, 2020 and the country-wide testing capacity for performing COVID-19 polymerase chain reaction (PCR) testing was established. Moreover, mass COVID-19 public awareness and educational campaigns were launched on multiple media platforms, including electronic, print and social media. Messages were developed to promote hand hygiene, social distancing, use of masks and PPE, environmental cleaning and disinfection, and to provide information on the disease itself. Helplines were also established for public facilitation. Additionally, to limit the importation of COVID-19 cases from neighboring countries, borders were shut down and screening of passengers at key airports and land crossings, along with establishment of information desks at all international airports and border crossings were initiated.

Early detection

Enhanced Surveillance and Screening

A key aim of surveillance is the early detection of an epidemic, to allow for rapid implementation of containment measures which are pivotal for the control and containment of infection. In recognition of this, the Federal M/o NHSR&C quickly identified gaps in active case finding and worked with the WHO to update and disseminate COVID-19 case definition and investigation protocols. Within each health facility, focal persons for surveillance were trained to identify and map COVID-19 cases. An 'Integrated Disease Information Management System' (IDIMS) was developed which linked the provincial surveillance systems to the national repository for all COVID-19-related data in Pakistan. Daily and weekly situation reports on COVID-19 were developed, and advanced data analyses were conducted to model disease projections and identify hotspots of infection [12]. In addition to screening all incoming passengers at major airports and other points of entry (land and sea ports), protocols were established for quarantining ill passengers and transporting them to health facilities and/or specially established quarantine centers.

Contact Tracing

The aim of contact tracing is to control the spread of infectious diseases through a combination of conventional methods, i.e., through tracking all people who came in physical contact with a patient, as well as through newer approaches that “track and trace” suspected individuals by using automation and sophisticated virtual applications [16]. Pakistan’s military played a pivotal role in tracing and tracking suspected COVID-19 case contacts. The operation was centrally located in Islamabad, and the provincial arms of the system carried out fieldwork. In addition, the M/o NHSR&C mobilized health frontline staff, including lady health workers (LHWs) and polio program staff to support contact tracing [17]. These frontline workers traced individuals returning from abroad and encouraged them to self-isolate, while creating awareness about prevention and containment among communities. Since LHWs and polio staff were already well-immersed in the community and trained for door-to-door campaigns, the approach worked well in the country’s fight against the fast-spreading virus [17].

Laboratory Testing Facilities

Pakistan's overall testing rate has remained low when compared to global testing rates. In January 2020, during the initial phase of the pandemic, Pakistan did not have COVID-19 testing facilities in the country and sent out samples to China, Japan, and the Netherlands for testing, which resulted in time lag and delays. However, the National Reference Public Health Laboratory quickly acquired the requisite capability for COVID-19 diagnostics in Pakistan, and soon after established multiple testing facilities across Pakistan that could perform real-time PCR testing for COVID-19 [18]. Pakistan’s total testing capacity in July increased to more than 46,500 tests per day, with a network of over 110 COVID-19 laboratories (Table 2). Unfortunately, the country utilized only a fraction of its testing capabilities due to financial constraints, and the unwillingness of people with mild COVID-19 symptoms to get tested due to the fear of being stigmatized and ostracized [18].

**Table 2:** Healthcare interventions were scaled up in Pakistan, March 2020 to September 2021. PCR: polymerase chain reaction

	MAR 2020	Jun 2020	SEP 2020	DEC 2020	MAR 2021	Jun 2021	SEP 2021
Labs with PCR	19	83	118	144	170	171	171
Testing capacity/day	6,584	30,362	47,980	56,219	61,577	78,055	77,479
Tests performed/day	935	29,085	32,933	39,695	40,369	46,145	44,889
Hospitals allocated for COVID Cases	449	820	735	626	631	639	640
Beds Allocated for COVID Patients	7,295	25,610	29,788	24,685	24,592	29,158	25,835
ICU beds allocated	2,441	3,090	5,577	6,368	6,368	6,368	3,476
Ventilators allocated	2,320	2,934	3,157	3,249	3,475	3,992	3.416

Containment and Control Strategies

Early detection of cases is insufficient to control the spread of COVID-19, unless containment and mitigation strategies such as quarantine, social distancing, travel restrictions, and lockdowns to limit crowding are implemented to prevent community transmission [19].

Quarantine and Travel Restrictions

Mandatory quarantine at a quarantine facility or through self-isolation is an attempt to involve the community and general population in implementing public health measures [20]. At an early stage of the epidemic, the government started establishing quarantine facilities and encouraged people to quarantine and self-isolate if they came in close contact with a confirmed COVID-19 patient. Overall, a total number of 294 quarantine facilities with 139,558 beds were established in the country, in addition to identifying approximately 500 designated hotels with 16,336 beds for quarantine purposes [12]. Furthermore, the M/o NHSR&C developed guidelines for all international travelers arriving in Pakistan and directed home quarantine of all contacts of a confirmed or suspected case of COVID-19.

Lockdown to Smart Lockdowns

To help contain the spread of COVID-19, various countries adopted a lockdown approach [21]. However, it negatively impacted poor and resource constraint countries such as Pakistan, leading to further financial and economic crises [22]. Pakistan enforced a complete lockdown in some of the major cities on March 21, 2020, for approximately two weeks. Thus, public transport, non-essential businesses, shopping malls, restaurants and public areas were shut down. All public gatherings were discouraged, and most provinces banned inter-city travel. Educational institutions, including schools, colleges, and universities remained closed and provided online education. On May 9, 2020, the provincial governments began phase-wise lifting of the national lockdown. In June 2020, Pakistan adopted the strategy of a 'smart lockdown', which involved identifying hotspots of infection through testing and contact tracing, and imposing a localized lockdown in focused areas with high disease spread [23]. This approach worked well to 'pursue a balance between people’s lives and livelihoods' and increase containment while also allowing businesses to remain open. Till September 2020, 904 smart lockdowns were imposed in Punjab, 26 in Sindh, 572 in Khyber Pakhtunkhwa, 29 in Azad Kashmir, 10 in Islamabad, and five in Gilgit-Baltistan [12]. Across the second to fourth waves of the epidemic in the country, similar smart lockdown approaches were implemented, with good dividends reaped with regards to the containment of the epidemic in the country.

Public Awareness Strategies to Control COVID-19 Spread at a Local Level

While a number of advisories, prevention guidelines and information materials were developed nationally, the local governments leveraged community networks and influencers to lead social and behavior change at the community level to ensure that preventive health and hygiene practices were being followed. Information on hand hygiene, social distancing, environmental cleaning, use of PPE including masks, and standard operating procedures (SOPs) on transmission precautions were widely disseminated through traditional communication channels such as TV, radio and print media, as well as through social media. The Ministry of Information Technology and Telecommunication, with the National Information Technology Board (NITB), developed a 'COVID app' with built-in features of self-assessment, radius alert, and pop-up notifications reminding people of maintaining their personnel hygiene [24]. A '*Sehat Tahaffuz*' (meaning Preserving Health) helpline was launched which provided people with health-related information. A 'COVID-19 telehealth portal' was also established where hundreds of doctors signed up and volunteered their time for a free tele-consultation with patients. The NCOC also launched a complaint line based on the notion of crowdsourcing, to notify 'COVID-related violations' by the general public. Through this complaint line, all citizens were provided with an opportunity to report violations of COVID-19 SOPs such as not wearing a mask, non-adherence to social distancing, or over-crowding of public places [25].

Mitigating Impact and Strengthening Health Care

Table 2 summarizes how healthcare interventions were scaled up in Pakistan to respond to the rising demand for healthcare and treatment due to COVID-19.

To cater to the growing need for COVID-19 testing, the government ramped up testing facilities and the training of laboratory personnel. The number of testing facilities increased from 19 in March 2020 to 118 in September 2020. The testing capacity also improved from 6,584 tests in March 2020 to nearly 48,000 tests in September 2020, and up to more than 82,000 tests per day by the last quarter of 2020. An increasing number of hospitals were designated for management of COVID-19 patients, and quality isolation COVID-19 wards were established. The number of COVID-19 treatment hospitals increased from 449 in March 2020 to 735 in September 2020, with nearly 30,000 beds available for COVID-19 patients. In addition, more than 6,000 ICU beds were allocated for seriously affected patients. Provincial governments also set up field hospitals to address the rising demands to treat affected patients. For example, in Sindh, the provincial government, in collaboration with the Pakistani army, set up multiple field hospitals across the province that could accommodate 10,000 patients. In Punjab, field hospitals were established in various locations including Rawalpindi and Jhelum. Moreover, NITB developed the 'Pak Neghayban' mobile phone-based digital application, which showed the availability of COVID-19 hospitals, ventilators, beds and testing facilities within various geographical areas [26]. Additionally, provincial health departments increased the hiring of doctors and health staff to address the escalating need for human resources. In Khyber Pakhtunkhwa (KP), approximately 1300 new doctors were hired, while in Punjab, 10,000 doctors and paramedics were recruited. By launching the 'Yaran-e-Watan' campaign in April 2020, the Pakistani government engaged overseas health professionals to provide telehealth consultation services to COVID-19 patients in Pakistan [26]. The issue of non-availability of PPE in the initial few days of the epidemic was quickly addressed by NDMA, by initially importing substantial quantity of PPE, and later scaling up its local production within Pakistan. The M/o NHSR&C launched 'We CARE' a national campaign for protecting and supporting frontline health workers with the aim of providing adequate PPEs to the health workers, orienting them on using various PPE items as per international standards, and creating an overall psycho-social environment of care [27]. The federal government also increased the local manufacturing of ventilators and testing kits to meet the increase in demand. The ventilators were manufactured by the Pakistan Engineering Council (PEC) in collaboration with the University of Engineering and Technology. The number of ventilators for COVID-19 patients was increased from 2,320 in March 2020 to nearly 3,500 since March 2021 onwards, which was enough to fulfill the projected needs [12].

Financial support

The containment measures introduced at the onset of the pandemic disrupted the economic situation in the country, taking the heaviest toll on the already disadvantaged and the poor. The government took various actions to support the people and businesses facing hardship as a result of the COVID-19 outbreak. A multi-sectoral economic relief package of PKR 1.25 trillion (8 billion USD) was approved to alleviate the economic impact of the COVID-19-related shutdown in the country [28]. The package included financial support for the daily-wagers and laborers, industrialists and exporters, farmers, and small and medium-sized enterprises (SMEs). It also aimed to provide relief to Utility Stores Corporation’s (USC) to help provide basic edible commodities at subsidized rates. The federal government also introduced an 'Ehsaas Emergency Cash Program' which supported more than 15 million families by providing financial assistance of PKR 12,000 (approx. 75 USD) [29]. With these interventions, the government aimed to address the dual challenge of tackling this extremely contagious and lethal pandemic, while at the same time trying to prevent a humanitarian and economic catastrophe.

Vaccination

Pakistan initiated registration of its population for COVID-19 vaccination in February 2021. Vaccination was initiated in early March 2021, and Table 3 below has the details for eligible age-wise population segments opening dates of registration for vaccination till September 2021. 

**Table 3:** Details for eligible age-wise population segments opening dates of registration for vaccination *The guidelines for immunizing children under 15 were recommended after November 2021

Age Group	Vaccination registration dates
65 and older	15^th^ Feb 2021
60 above	10^th^ March 2021
50-59 years	21^st^ April 2021
40-49 years	3^rd^ May 2021
30-39 years	16^th^ May 2021
18 plus	3^rd^ June 2021
15-17 years*	13^th^ September 2021

**Figure 3:**
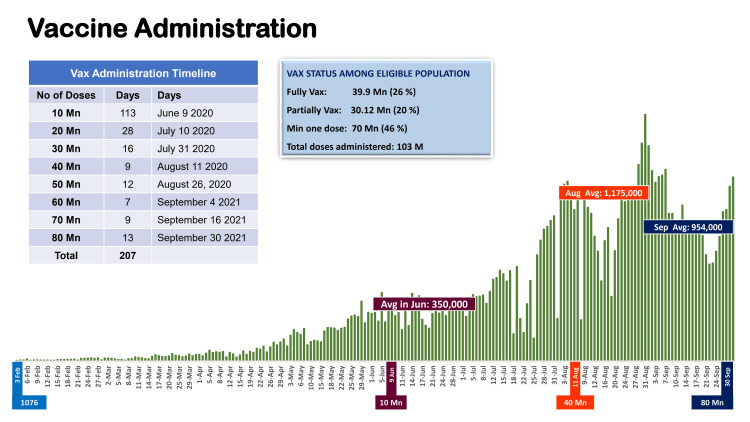
Vaccine administration in Pakistan across February 2021 to September 2021 This is an original figure

The vaccination process was a bit slower initially, but saw an exponential increase from June 2021 onwards. The first 10 million doses were administered in 113 days, the second 10 million in 28 days, the third 10 million doses were administered in 16 days and the last 10 million doses in only 12 days; with the health system administering more than 1 million doses on an average per day, since September 2021 (Figure 3).

As of September 30, 2021, Pakistan administered a total of 82.5 million doses, and fully vaccinated 29.2 million or about 20.05% of 15 years and above (total eligible population = 146 million). Some of the key achievements in this regard included: firstly, deployment across each district of the country and free-of-cost administration of 82.5 million doses (till September 30, 2021), which include AstraZeneca-Oxford vaccines, Sinopharm, Sinovac, CanSino, Sputnik-V, Moderna, PakVac-Cansino and Pfizer BioNTech (mRNA Covid-19 vaccine); secondly, expedient review of available literature, and emergency use approval (EUA) for seven vaccines in the country by the Drug Regulatory Authority of Pakistan (DRAP) (Sinopharm, CanSino, Sinovac, Sputnik V, Astra Zeneca, Pfizer and PakVac-Cansino); thirdly, permissions to the private sector for import and administration of vaccines with strict pricing control mechanism regulated through DRAP; fourth, established 770 COVID-19 vaccination centres (CVCs) and 22 mass vaccination centres across the country, which are being increased; and finally, packaging and production of in-country COVID-19 vaccines through the NIH.

## Conclusions

A number of key lessons can be learned from Pakistan’s response strategy, which could benefit countries and regions struggling to flatten the COVID-19 curve. Pakistan’s response to the first wave of the COVID-19 pandemic was consistent with the WHO’s suggested stages of epidemic response interventions, specifically, anticipation, early detection, containment and mitigation. An important lesson learned is that a successful epidemic response requires strong and decisive political leadership, that can oversee the development of a unified strategy based on evidence-informed approaches. A strong factor that contributed significantly to Pakistan’s effective COVID-19 response was the development of NCOC early in the outbreak, which allowed the federal, provincial and regional governments to develop a comprehensive strategy and coordinate a unified response against COVID-19. Strong national coordination is needed to avoid the implementation of diverse strategies that can cause confusion among the general population. The timeliness of the response and the quick implementation of response measures was another key strength of Pakistan’s COVID-19 strategy. Thus, factors such as coordinating and implementing a nationwide lockdown at a fairly early stage in the epidemic, quickly building healthcare capacity, and initiating public health awareness campaigns, and massive vaccination drive for the fifth largest population of the world, played an important role in mitigating the spread of the virus. Additionally, implementing measures such as international border closures, contact tracing and surveillance during the anticipation and early detection stages of the outbreak significantly prevented the importation of a large number of COVID-19 cases. The mobilization of the LHWs and polio program staff to support contact tracing strengthened the community-based response against the virus. Furthermore, the use of 'smart lockdowns' to shut down COVID-19 hotspots helped reduce virus transmission while ensuring that the interruption to livelihoods was reduced; this approach could also be useful for other countries with fragile economies. 

Pakistan is among the countries that responded well to the novel coronavirus, and as such was able to significantly reduce transmission and flatten the curve. The response was well coordinated and used an evidence-based approach that was backed up by national leadership and coordination. The crisis, however, is not yet over, and Pakistan could experience a resurgence of COVID-19 cases with more severe intensity. Until everyone has widespread access to a vaccine, which might not occur for some time, the nation has to continue implementing strong COVID-19 response measures to prevent future waves of COVID-19. The vaccination capacity of the country progressed well, but the vaccination process has faced challenges due to widespread hesitancy because of negative publicity mainly related to the safety profile, efficacy of vaccines and recurrence of infection after getting vaccinated. 
